# Clonal dynamics monitoring during clinical evolution in chronic lymphocytic leukaemia

**DOI:** 10.1038/s41598-018-37389-7

**Published:** 2019-01-30

**Authors:** Julia González-Rincón, Sagrario Gómez, Nerea Martinez, Kevin Troulé, Javier Perales-Patón, Sophia Derdak, Sergi Beltrán, Belén Fernández-Cuevas, Nuria Pérez-Sanz, Sara Nova-Gurumeta, Ivo Gut, Fátima Al-Shahrour, Miguel A. Piris, José A. García-Marco, Margarita Sánchez-Beato

**Affiliations:** 1 Lymphoma Research Group, Medical Oncology Department, Instituto de Investigación Sanitaria Puerta de Hierro-Segovia de Arana, Madrid, Spain; 2 Centro de Investigación Biomédica en Red de Cáncer (CIBERONC), Madrid, Spain; 3 Pathology Department/Translational Hematopathology Group, Hospital Universitario Marqués de Valdecilla/IDIVAL, Santander, Spain; 4 Bioinformatics Unit, Spanish National Cancer Research Centre (CNIO), Madrid, Spain; 5 CNAG-CRG, Centre for Genomic Regulation (CRG), Barcelona Institute of Science and Technology (BIST), Barcelona, Spain; 6 Universitat Pompeu Fabra (UPF), Barcelona, Spain; 7 Hematology Department, Hospital Universitario Puerta de Hierro-Majadahonda and Instituto de Investigación Sanitaria Puerta de Hierro-Segovia de Arana, Madrid, Spain; 8 Pathology Department, Hospital Fundación Jiménez Díaz, Madrid, Spain

## Abstract

Chronic lymphocytic leukaemia is the most prevalent leukaemia in Western countries. It is an incurable disease characterized by a highly variable clinical course. Chronic lymphocytic leukaemia is an ideal model for studying clonal heterogeneity and dynamics during cancer progression, response to therapy and/or relapse because the disease usually develops over several years. Here we report an analysis by deep sequencing of sequential samples taken at different times from the affected organs of two patients with 12- and 7-year disease courses, respectively. One of the patients followed a linear pattern of clonal evolution, acquiring and selecting new mutations in response to salvage therapy and/or allogeneic transplantation, while the other suffered loss of cellular tumoral clones during progression and histological transformation.

## Introduction

Chronic Lymphocytic Leukaemia (CLL) is the most prevalent leukaemia in Western countries. It is an incurable disease characterized by a highly variable clinical course that results from expansion of clonal CD5 + B-lymphocytes growing in bone marrow (BM), blood, lymph nodes (LN) or other lymphoid compartments. In the last years, multiple studies have reported that CLL disease is not just heterogeneous between patients but also individual CLL samples are genetically heterogeneous and contain subclonal cellular populations^1–3^. It is broadly accepted that this diversity evolves under selective treatment pressures, such as chemotherapy, that favor selection of resistant clones that emerge after treatment^1,4^. Two major patterns for clonal evolution have been described: linear and branched evolution. In the linear evolution model, a linear sequence of mutational events takes place within a single clone while branched evolution presents spatial and temporal heterogeneity and two or more clone could coexist and evolve in parallel^5^.

CLL is an ideal model for studying clonal heterogeneity and dynamics during cancer progression, response to therapy and/or relapse because the disease usually develops over several years, it could affect different haematological compartments and its presentation is fairly heterogeneous among patients^3,6,7^. Here we report an analysis by deep sequencing of sequential samples taken at different times from the affected organs of two patients with 12- and 7-year disease courses, respectively.

## Results

### Whole exome sequencing in index samples

Patient 1 (P1) was a 50-year-old male diagnosed with stage A/II CLL in 2000 (see Patient 1 description in “Methods” section). He was CD38-negative, ZAP70-positive, had unmutated IgHV, and a normal karyotype with an 11q22-23 deletion (ATM) in 67% of interphase nuclei, as revealed by fluorescence *in situ* hybridization (FISH) analysis. In March 2004 the patient was treated with fludarabine achieving partial remission (PR); two years later, in March 2006, he came to our institution with symptomatic disease, generalized lymphadenopathy and splenomegaly. Before he started treatment with FCR (fludarabine, cyclophosphamide, rituximab) plus rituximab maintenance for 6 months, we collected sample P1.1 of peripheral blood mononuclear cells (PBMCs). In September 2012 (aged 61 years, 12 years after diagnosis) the patient died of disease progression and samples were obtained from different compartments: P1.13 (PBMCs), P1.14 (lymph node) and P1.15 (spleen). Whole exome sequencing (WES) was performed in these index samples as well as in DNA from oral mucosa (P1.N). Our analysis identified 46 non-synonymous variants (non-sense, missense, frameshift and splicing) as being present in at least one sample.

Patient 2 (P2) was a 62-year-old male diagnosed with stage B/II CLL in September 2006 (see Patient 2 description) the patient presented symptomatic disease. The first sample (P2.1, PBMCs) was taken in January 2007, and it was CD38-negative, ZAP-70-positive, IgHV unmutated, and had a normal karyotype with deletions of 11q22-23 and 13q14 detected by FISH in 50% and 25% of interphase nuclei, respectively. In March 2008 was treated with 6 cycles of FCR, and rituximab maintenance for 3 years (REM [Rituximab in maintenance] clinical trial) and in October 2012 (P2.4, PBMCs), P2 rapidly developed an increase in lymphocytosis with generalized lymphadenopathy and was treated with anti-CD37 (TRU-016) plus bendamustine and attained PR. In July 2013, a Richter transformation was diagnosed in a lymph node (LN) biopsy (P2.5), the patient was treated with salvage chemotherapy plus bortezomib, but had no clinical response and died in January 2014, aged 68, 7 years after diagnosis. WES was performed in these index samples and oral mucosa (P2.N) and we identified 32 non-synonymous variants present in at least one of them.

### Clonal heterogeneity and evolution

With the whole set of non-synonymous variants identified by WES, we designed a custom panel to analyse every available sample by deep sequencing (median coverage 4125×), including the index samples (Table S1). This, together with the karyotype and FISH information, allowed us to follow the genetic changes on 13 points over 6 years in P1 and in 5 samples over 7 years in P2.

#### Patient 1

**Figure 1:**
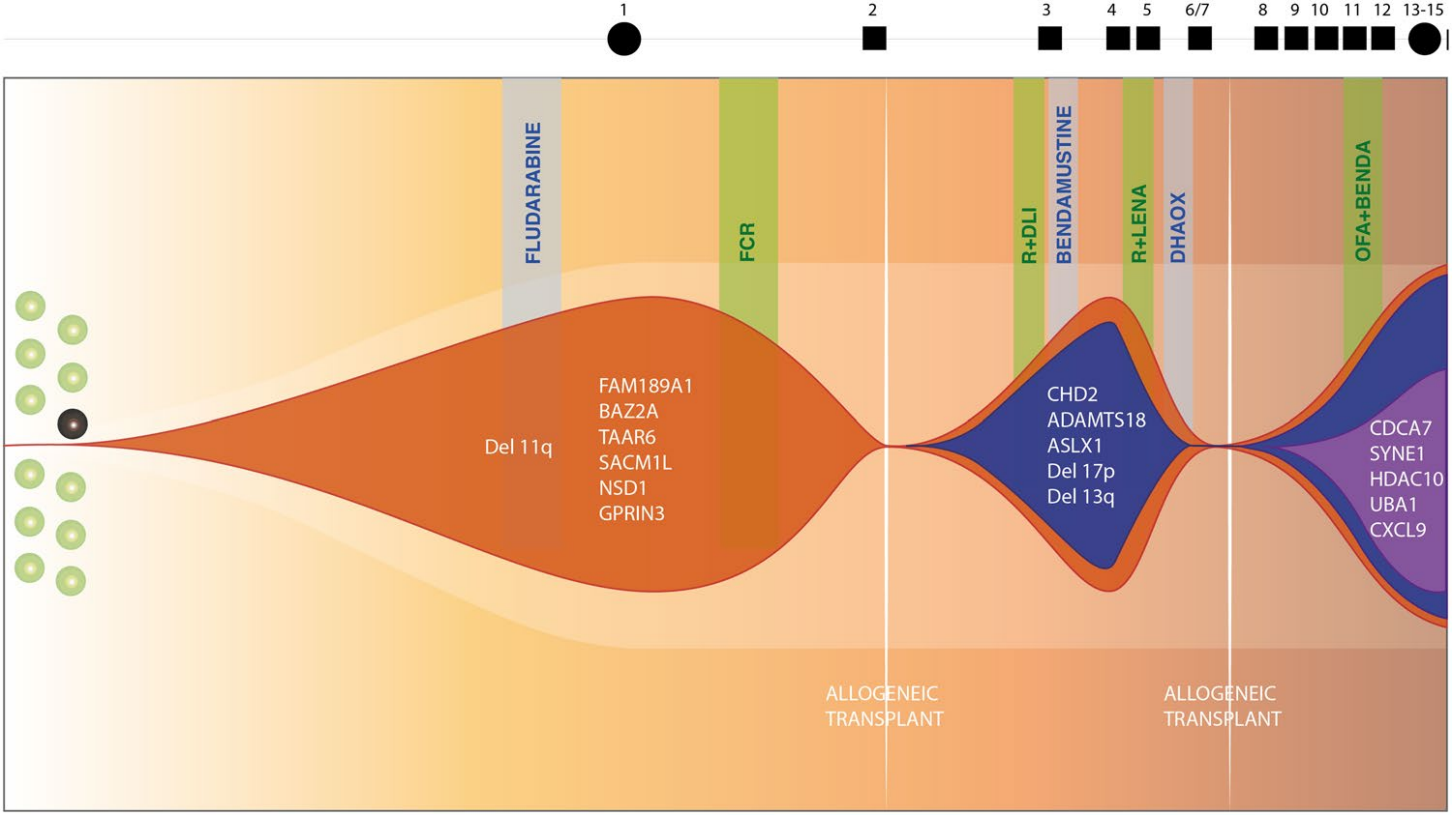
Schema of the inferred tumour clonal evolution path of the CLL patient 1. Sequential acquisition of somatic alterations in patient 1 leads to its genomic diversification. Exposure to successive chemotherapy, immunochemotherapy and allogeneic transplants would be associated with acquisition of new alterations and may profile the CLL landscape. FCR: fludarabine, cyclophosphamide, rituximab; R + LENA: rituximab plus lenalidomide; OFA-Benda: ofatumumab-bendamustine; DLI: donor lymphocyte infusion; DHA0X: high-dose cytarabine and oxaliplatin.

In P1, FISH revealed an 11q22-23 (*ATM*) deletion in 90% of tumoral cells when diagnosed in September 2000 (see Patient Description). In March 2004, P1 was treated with fludarabine achieving PR; two years later, in January 2006, P1 showed symptomatic disease with lymphadenopathy and splenomegaly and was treated with FCR and rituximab maintenance. In sample P1.1 (March 2006, before treatment with FCR)), *ATM* and 17p13.1 (*TP53*) deletions were detected in 85% and 3% of cells, respectively, as well as somatic mutations in seven genes (*TP53*, *FAM189A1, SACM1L, TAAR6, GPRIN3, NSD1* and *BAZ2A*) with variant allelic frequencies (VAFs) ranging from 17% to 50% (Table S2 and Fig. S1A–C in SI). In June 2007 the patient received a non-myeloablative allogenic stem cell transplant (SCT). Later on, in February 2009, the disease progressed and the patient was treated with rituximab and donor lymphocyte infusion, attaining PR; in the sample analyzed in September 2009 (P1.3) we were able to detect new sub-clonal mutations in *CHD2*, *ASXL1* and *ADAMTS18*, among other genes, with VAFs ranging from 3% to 6% (Table S2 and Fig. S1A,B and C in SI). At this point, 11q22-23, 17p13.1 and 13q14 deletions (66%, 23% and 30% respectively) and *MYC* amplification (15%) were detected by FISH. P1 was treated with R-bendamustine (September 2009) without response, and subsequently was given R-lenalidomide (March 2010, P1.4), which yielded a short-lived response, with the patient progressing in October 2010. The patient rapidly developed a generalised growth of lymph nodes and lymphocytosis that were refractory to two subsequent lines of treatment (R-bendamustine and R-lenalidomide). Mutational data validated that tumoral cells did not respond to treatment with R-bendamustine (median VAF increased from P1.3 to P1.4) and only partially to R-lenalidomide (slight decrease in VAFs in sample P1.5, September 2010). In February 2011 (P1.6), cytogenetic and mutational data indicated an increase in the tumoral cell population. The patient was subjected to salvage chemotherapy with four cycles of dexamethasone, high-dose cytarabine and oxaliplatin, which produced a PR (P1.7), and consolidated in August 2011 with a second non-myeloablative allogeneic SCT (sample P1.8 taken before transplant). The same mutations, although with lower VAF than in P1.6, were detected in P1.7, reflecting the PR to salvage chemotherapy. Cytogenetic data were consistent with mutation changing patterns provided by the sequencing analysis data. In February 2012, after treatment with salvage chemotherapy and allogeneic SCT the patient progressed and sample P1.9 was found to have new mutated genes (*CDCA7*, *SYNE1*, *HDAC10*, *UBA1*, *CXCL9*, among others), with VAFs from 7% to 15%. FISH revealed deletions of 11q22-23 (in 95% of cells), 13q14 (57%) and 17p13 (76%), in addition to a complex karyotype (see Patients’ description in the Methods section). During the period from February to September 2012 (exitus), P1 was treated with ofatumumab-bendamustine without clinical response; samples P1.10 to P1.13 were also sequenced, and showed increase in the VAFs of the mutations and in the percentage of cells with cytogenetic alterations (Fig. 1, Table S2A,B and Fig. S1A–C in SI).

**Figure 2:**
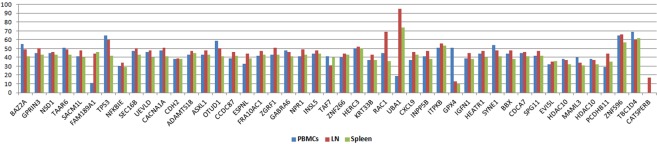
Variant Allelic Frequency of the non-synonymous somatic mutations in peripheral blood lymphocytes (PBMCs), lymph node (LN) and spleen at the end of the disease from Patient 1.

We also analysed the differences in genetic alterations in samples from different anatomical locations, LN, spleen and PBMCs at the end-stage of the disease (P1.13-15), and found that the three compartments shared almost the same variants, although some had different VAFs (Figs 2, S1D and Table S2A,B in SI), such as *CASTPERB*, which was exclusively mutated in LN or *GPX4* with higher VAF in PBMCs.

#### Patient 2

**Figure 3:**
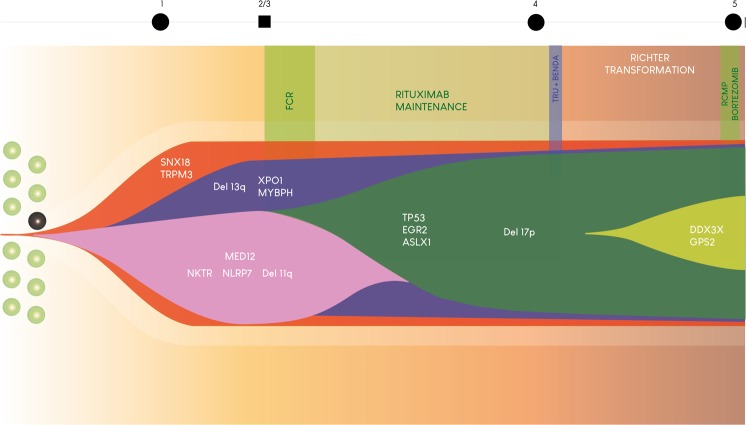
Schema of the inferred tumour clonal evolution path of the CLL patient 2. CLL therapy could induce novel mutagenesis and/or accelerate clonal evolution by “killing” a clone and selecting other containing mutations in genes that might confer resistance. Sustained treatment with targeted antigen-specific immunotherapy with anti-CD20 and subsequent anti-CD37 monoclonal antibodies might interfere with stem cell plasticity and provoke tumor escape through clonal evolution with acquisition of additional genetic alterations responsible for histological transformation. FCR: fludarabine, cyclophosphamide, rituximab; TRU-Benda: TRU-016-bendamustine; RCMP: rituximab, liposomal doxorubicin and prednisone.

P2 showed at three months after diagnosis (sample P2.1, January 2007) concomitant 11q22-23 and 13q14 deletions in 50% and 25% of tumoral cells, respectively. The landscape of somatic mutations in this patient was consistent with the existence of two clones derived from an ancestral one with mutations in *SNX18* and *TRPM3* (VAF of 50%, meaning around 100% of cells) (Table S3A,B and Fig. S2 in SI). At diagnosis, one clone encompassed the 11q22-23 deletion and mutations in *MED12*, *NLRP7* and *NKTR* (50% of cells), whose VAF decreased over the course of the disease; the second clone featured the 13q14 deletion (in 25% of the cells) and mutations in *XPO1*, among others, whose median VAF increased over time, as may be seen by comparing the patterns of mutations in samples P2.1 and P2.2. No treatment was given to P2 until March 2008 when he was treated with FCR and rituximab maintenance for 3 years, in the context of a clinical trial, and achieved complete response (samples P2.2 [PBMCs] and P2.3 [bone marrow] were taken in February 2008, before treatment). In September 2012 (sample P2.4) the patient developed general lymphadenopathy and exhibited a complex karyotype along with 11q22-23 (9%), 17p13 (55%) and 13q14 deletions (63%), and *MYC* amplification (19%) detected by FISH; a new tumoral clone with mutations in *TP53*, *EGR2* and *ASXL1* emerged while the clone with 11q22-23 deletion was almost completely lost, in parallel with an increase of the subclone with an 13q14 deletion and *XPO1* mutation. The patient was treated with TRU-016 (anti-CD37) and bendamustine (October 2012) achieving PR. In July 2013, P2 developed a generalized lymphadenopathy along with malaise, weight loss, daily fever and night sweats. A Richter transformation was diagnosed after a LN biopsy (sample P2.5; see Patient Description in SI). In this sample, neither the 11q22-23 deletion, nor the variants in *MED12*, *NLRP7*, *NKTR*, *PTPRG* or *PRDM6* could be detected, but new mutated genes emerged, *DDX3 X * and *GPS2* being the most relevant. In the case of the mutational landscape of PBMCs (P2.2) and bone marrow (P2.3), the same pattern and VAFs in mutated genes were found. Although samples P2.4 and P2.5 came from different organs (PBMCs and LN, respectively), a Richter transformation occurred between the two time-points. It was not possible to determine whether these differences were due to the transformation (although this was the most likely cause) or to the different organs involved, due to the absence of a PBMC sample obtained simultaneously with the LN sample, because the patient did not have lymphocytosis (Fig. 3, Table S3A,B and Fig. S2 in SI). Patient was treated with salvage chemotherapy and bortezomib with two cycles without clinical response and died in January 2014 of disease progression.

## Discusion

In this study, we described results from sequencing sequential samples taken at different time-points of disease evolution from two CLL patients with 12- and 7-year disease courses.

Patient P1 followed a linear pattern of clonal evolution, acquiring and selecting new mutations in response to salvage therapy and/or allogeneic SCT (Fig. 1). The founder alteration was the 11q22-23 deletion and the acquisition of the *TP53* biallelic alteration, one of the clearest predictive factors of treatment resistance, appeared to be the inflection point. The successive treatments received by P1 not only failed to eliminate the initial clones but also contributed linearly to, or at least did not prevent, the acquisition of new alterations, which did not respond to any subsequent treatment. This fact was more evident after the two non-myeloablative allogenic SCT, when a higher number of new mutations appeared (Table S2A). After the first SCT, mutations in *CDH2*, *ADAMTS18* and *ASXL1* were detected with low VAFs (P1.3) that increased during disease evolution (P1.4 and successive samples). After the second one, genes such as *ITPKB*, *IGFN1*, *SYNE1*, *CDCA7* and *HDAC10*, among others, were also found mutated, probably giving a survival advantage to the tumoral clone.

When we compared the mutational landscape in PBMCs, LN and spleen, we did not detect any significant differences between the three compartments; previous studies analysing different parts of the same tumour or single cells, have described differences in their mutational landscapes^8,9^. In our case, we were not able to perform this type of single cell or spatial analysis, and, therefore, putative minor clones, with low VAFs, may be underrepresented. The clones we have detected were similar in the three compartments and may be attributed to predominant clones with a broader somatic mutational landscape.

We draw attention to two points regarding patient P2: the displacement of the clone with 11q22-23 deletion and mutations in driver genes such as *MED12* by that with the 13q14 deletion and mutation in *XPO1*, and the presence of a new tumoral clone that emerged after FCR-Rm treatment, with a *TP53* biallelic alteration, *MYC* amplification and mutations in *EGR2*, *AXL1* and *RPS15* (Fig. 3). *XPO1* mutation has been described as a recurrent and initiating event in CLL and other B cell-derived neoplasias^10,11^. *EGR2* mutations have been associated with clinically aggressive CLL^12,13^. Eventually, after anti-CD37 plus bendamustine therapy, the Richter transformation was diagnosed. The *TP53* biallelic alteration is associated with a higher risk of transformation as well as *NOTCH1* activation and *MYC* abnormalities^14,15^. A previous study identified mutations in *DDX3X*, *RPS15* and *GPS2* (which were all mutated in P2) as part of a driver gene profile associated with 17p deletion in CLL^16^. RNA processing and splicing alterations play central roles in this patient: four genes (*MED12*, *XPO1*, *DDX3X* , *RPS15*) involved in these biological processes were found to be mutated, as well as chromatin remodelling genes (*PRDM6, ASXL1, MSX2*) (for a review see^17^).

*ASXL1* was found mutated in the two patients after FCR treatment. *ASXL1* mutations have been associated with shorter overall survival and worse response to immunosuppression^18^. A case report of a CLL patient^19^ also identified a mutation in *ASXL1* gene after FCR treatment, and other studies focused on acute myeloid leukaemia and myelodysplastic syndrome have identified mutations in *ASXL1* after treatment with chemotherapy^20–22^. We hypothesize that the appearance of *ASXL1* mutations in the two patients could be a consequence of the CIT received by both patients.

In summary, this study provides complementary information about clonal evolution and heterogeneity in CLL. We show here two different patterns of clonal dynamics, P1 acquiring new alterations at relapse, after allogeneic SCT and salvage chemotherapy, and P2 loss cellular tumoral clones after treatment and during progression. We also hypothesize that there might be two mechanisms involved in the pathogenesis of non-germinal centre DLBCL transformation in CLL. First, cell-cycle dysregulation induced by acquired *MYC* rearrangements, along with the impairment of the DNA damage response through acquired *TP53* loss, may play a role in the transformation event. Second, sustained treatment with targeted antigen-specific immunotherapy with anti-CD20 and subsequent anti-CD37 monoclonal antibodies might interfere with stem cell plasticity and provoke tumour escape through acquisition of additional genetic alterations, responsible for histological transformation, some of them associated to 17p deletion in CLL.

## Methods

### Patients Description

This study was approved by the Hospital Universitario Puerta de Hierro-Majadahonda institutional ethical committee. Patients provided informed consent in accordance with local institutional review board requirements and the Declaration of Helsinki before enrolment. All research methods were performed in accordance with Institutional Guidelines and regulations. Treatment protocols were according to the Haematology Department guidelines and patient 2 also participated in two research phase II clinical trials (REM: EudraCT No.: 2007-002733-36 and Otlertuzumab plus Bendamustine: NCT01188681) approved by the Institutional ethics committee.

Patient 1, a physically fit 50-year-old male was diagnosed in September 2000 with stage A/II CLL, with splenomegaly of 4 cm below the left costal margin (b.l.c.m.), a lymphocyte count of 112,000/µL, a haemoglobin count of 14.5 g/dl, and a platelet count of 150,000/µL. He was CD38-negative, ZAP70-positive, had unmutated IgHV, and a normal karyotype with an 11q22-23 del (ATM del in 67% of interphase nuclei), as revealed by fluorescence *in situ* hybridization (FISH).

In May 2004, 45 months after diagnosis, the patient presented with increasing fatigue, a lymphocyte doubling time of 6 months and splenomegaly of 6 cm b.l.c.m. He was treated with fludarabine (25 mg/m^2^ × 5 days × 6 cycles) and achieved partial remission.

He presented at 21 months since first treatment (64 months from diagnosis) with symptomatic disease, generalized lymphadenopathy and splenomegaly of 4 cm b.l.c.m. (Sample P1.1, February 2006) and in March 2006 (67 months from diagnosis) was treated with FCR (fludarabine, cyclophosphamide, rituximab) plus rituximab maintenance for 6 months, achieving complete clinical remission, and was consolidated with a non-myeloablative allogeneic stem cell transplant (SCT) of HLA-identical unrelated adult donor in June 2007 (82 months after diagnosis) (Sample P1.2, before transplant).

The patient relapsed 20 months after transplantation (102 months, February 2009) with rapidly progressive lymphocytosis and generalized lymphadenopathy of 2–4 cm and retroperitoneal adenopathy of 6 cm, along with acquisition of a 17p1.3 deletion, as revealed by FISH, and a *TP53* mutation, as identified by cDNA Sanger sequencing (inframe deletion: c.376_396del21; p.Y126_K132delYSPALNK). He was treated with rituximab and increasing monthly doses of donor lymphocyte infusion (4 cycles), and achieved partial remission (PR). The patient progressed in October 2010, developing rapidly growing generalised lymph nodes and lymphocytosis that were refractory to two subsequent lines of treatment (R-bendamustine and R-lenalidomide).

In February 2011 (126 months after diagnosis), the patient received four cycles of salvage chemotherapy with dexamethasone, high-dose cytarabine and oxaliplatin, achieving PR, and was consolidated in August 2011 with a second non-myeloablative allogeneic SCT of HLA-identical unrelated cord-blood. Patient remained in partial remission until February 2012 (138 months after diagnosis), when progressed with a 7-cm bulky retroperitoneal lymphadenopathy and was treated with ofatumumab-bendamustine for 3 cycles, but without clinical response. In September 2012, the patient died of disease progression without histopathological evidence of Richter transformation.

Patient 2, a physically fit 62-year-old male was diagnosed in September 2006 with stage B/II CLL with generalized lymphadenopathy of 1.5–2.0 cm without hepatomegaly or splenomegaly, a lymphocyte count of 16.750/µL, a haemoglobin count of 14.8 g/dl and a platelet count of 159,000/µL. His sample was CD38-negative, ZAP-70-positive, IgHV unmutated, and had a normal karyotype with deletions of 11q22-23 (*ATM*) and 13q14 (D13S319) detected by FISH in 50% and 25% of interphase nuclei, respectively.

The patient was treated in March 2008 (15 months after diagnosis) with 6 cycles of FCR, and rituximab maintenance for 3 years (REM clinical trial), and he achieved a complete response.

In October 2012 (70 months after diagnosis) the patient developed rapidly increasing lymphocytosis with generalized lymphadenopathy of 3-4 cm and molecular cytogenetic studies showed a complex acquired karyotype [45–46, XY, −10, del(11)(q14;q21), del(11)(q11), del(13)(q14;q21), i(17)(q10)] with deletions of 17p13 (p53) (55%), 13q14 (63%), C-MYC amplification (19%) and a notably low percentage of ATM deletion (9%) in the 11q22-23 region. The patient was treated with anti-CD37 plus bendamustine for 6 cycles (clinical trial) and achieved partial remission. In July 2013 (79 months) presented with rapidly growing cervical and axillary lymphadenopathy of 3–5 cm along with progressive malaise, weight loss, night sweats and fever with no documented active infection. A lymph node biopsy showed Richter transformation into a diffuse large B cell lymphoma (DLBCL) (Ki67: 70%, CD20+ high, CD5−, CD10−, BCL-6−, MUM-1+, FOXp1+, CD38+ and EBERs−). The bone marrow had no infiltration by DLBCL, but showed diffuse infiltration of small and medium size lymphocytes. CD20+, CD5+, CD23+, BCL2+ and TP53 overexpression and Ki67+ (40%). FISH data showed 17p13 deletion (75%), homozygous 13q14 deletion (83%), and *CMYC* amplification/translocation (19%). P2 was treated with salvage immunochemotherapy (rituximab, liposomal doxorubicin and prednisone) and bortezomib for 2 cycles without clinical response and died in January 2014 (86 months after diagnosis), 7 months after diagnosis of transformation to non-germinal centre DLBCL with the same IgHV mutational pattern as the original CLL clone.

### Fluorescence *In Situ* Hybridization (FISH)

Cytogenetic aberrations were analysed by FISH with the VysisCLL FISH Probe Kit, following the manufacturer’s recommendations for detecting deletions of TP53 (17p13.1), ATM (11q22.3), D13S319 (13q14.3), MYC rearrangements/amplification (8q24.12-q24.13) and gain of the D12Z3 sequence (trisomy 12) in peripheral blood specimens from CLL patients.

Cut-off values for a positive FISH result were 3% and 10% for gains and deletions, respectively. Routine FISH testing is generally performed for patients with CLL prior to starting treatment. Follow-up FISH testing is not routinely performed at defined intervals but may be requested by the treating physician when there is disease progression and treatment is considered.

### Samples

Fifteen samples were collected from peripheral blood of Patient 1 between diagnosis and exitus (P1.1 to P1.13) as well as biopsies from lymph node (P1.14) and spleen (P1.15) from necropsy and buccal mucosa (P1.N). We analysed three samples from peripheral blood mononuclear cells of Patient 2 (P2.1, P2.2 and P2.3) one from bone marrow (P2.4), a lymph node (P2.5) and buccal mucosa for germinal DNA (P2.N).

DNA was obtained from peripheral blood mononuclear cells separated by Ficoll (Rafer, Zaragoza, Spain). Tumour cell purity was calculated based on the CD19/CD5 ratio, measured by FACS.

DNA was extracted with DNAzol Genomic DNA Isolation Reagent (Molecular Research Center, OH, USA) according to the manufacturer’s instructions. The quality and quantity of purified DNA was assessed by both fluorimeter (Qubit, Invitrogen, MA, USA) and gel electrophoresis.

### Whole-Exome Sequencing

Whole-exome sequencing (WES) was performed in DNA from mononuclear cells of index samples (P1.1, P1.13, P1.14, P1.15 and P1.N from Patient 1, and P2.1, P2.4, P2.5 and P2.N from Patient 2).

Whole-exome capture and sequencing were performed at the Spanish National Centre for Genomic Analysis (CNAG, Barcelona, Spain). Tumour and germline DNA were enriched for coding regions in the genome using the SureSelect-XT Human exon capture 50 Mb kits V4 and V5 (Agilent Technologies, Santa Clara, CA, USA). According to the manufacturer’s protocol, these include the coding exons of c.20,000 genes, covering > 50 Mb of the genome. Paired-end sequencing, resulting in 76 bases from each end of the fragments, was performed using a HiSeq. 2000 Genome Analyzer (Illumina Inc., San Diego, CA, USA). Targeted NGS data was demultiplexed with HiSeq Software (Illumina). Base calling and quality control were carried out with the Illumina RTA sequence analysis pipeline (Illumina) (Supplementary Table S4).

We performed two parallel pipelines for alignment and variant calling. In the first, the sequence reads were trimmed until the first base was read with a quality of more than 10. Mapping to human genome build hg19 (GRCh37) was done with the GEM^23^, allowing up to four mismatches. Reads not mapped by the GEM (approximately 4%) were subjected to a final round of mapping with BFAST^24^. Results were merged and only uniquely mapping non-duplicate read pairs were used for further analyses. The SAMtools suite^25^, with default settings, was used to call single nucleotide variants and short insertions and deletions. Identified variants in regions with low mappability, a read depth of <10 or a strand bias with a value of P < 0.001 were filtered out. Variant annotation and effect prediction were performed with Annovar^26^ and snpEff^27^, including information from the Single Nucleotide Polymorphism Database (dbSNP, build 135)^28^, the 1000 Genomes Project, the Exome Variant Server (NHLBI GO Exome Sequencing Project, Seattle, WA, USA; http://evs.gs.washington.edu/EVS/) and an internal database of sequence variants identified in a set of more than 100 control samples. To compare tumour and normal cells, the significance of Fisher’s exact test was calculated for positions with different genotypes in the two samples based on alternative allele frequencies.

For the second pipeline, quality was assessed using the FASTQC tool (http://www.bioinformatics.babraham.ac.uk/projects/fastqc/), and alignment performed using Burrows-Wheeler Aligner^25^ using GRCh37/hg19 assembly. The samples were realigned with the GATK tool^29^ to improve variant calling, which was done with Mutect 1.1.7^30^. Annotation was carried out with Variant Effect Predictor (VEP)^31^.

The sequence data have been deposited in the Sequence Read Archive at http://www.ncbi.nlm.nih.gov/sra/, with accession number (SUB2978993).

### Target Sequencing

Based on mutations identified by WES, a TruSeq Custom Amplicon v1.5 panel was designed. The target regions are provided in Table S1 in SI. The probes for this targeted custom panel were designed with DesignStudio (Illumina) and consisted of 266 amplicons with an average size of 180 bp and a cumulative targeted region of 20 kb. Polymorphisms were avoided in the design of primers.

DNA target enrichment was performed using the protocol described in the TruSeq Amplicon—Cancer Panel Library Preparation Guide (March 2016; Illumina). The total amount of input DNA for capture ranged from 30 to 100 ng. After preparation, indexing and bead purification, the libraries were quantified by Qubit analysis to assess successful enrichment and amplification. Libraries were then normalized on beads and pooled for sequencing in accordance with the TruSeq Amplicon protocol. The pooled libraries were paired-end (2 × 151), and sequenced with V2 chemistry on a MiSeq instrument (Illumina), as described in the manufacturer’s protocol. Alignment and variant calling were performed using MiSeq reporter and variants identified in previous analyses were verified by visualization in IGV tool^32^ (Integrative Genomics Viewer).

Somatic single nucleotide variants (SNVs) identified by deep sequencing were used as the input into PyClone (v0.13.0) to identify those with similar VAFs and to infer the different clones and their patterns of evolution^33–35^.

## Supplementary information

Supplementary figures

Sup Table S1

Sup Table S2A

Sup Table S2B

Sup Table S3A

Sup Table S3B

Sup Table S4
