# Supplementary material for: Clonal dynamics monitoring during clinical evolution in chronic lymphocytic leukaemia
Source: Sci Rep. 2019 Jan 30;9:975. doi: 10.1038/s41598-018-37389-7 (PMC6353992; doi:10.1038/s41598-018-37389-7)
Supplement: Supplementary file 1 — Supplementary figures [file 41598_2018_37389_MOESM1_ESM.pdf]

Supplementary Figures

Clonal dynamics monitoring during clinical evolution in chronic lymphocytic leukaemia

Julia González-Rincón[1,2], Sagrario Gómez[1], Nerea Martinez[2,3], Kevin Troulé[4], Javier Perales-Patón[4], Sophia Derdak[5,6], Sergi Beltrán[5,6], Belén Fernández-Cuevas[7], Nuria Pérez-Sanz[7], Sara Nova-Gurumeta[7], Ivo Gut[5,6], Fátima Al-Shahrour[4], Miguel A. Piris[8,2], José A. García-Marco[7#, *], Margarita Sánchez-Beato[1#, *]

1. *Lymphoma Research Group, Medical Oncology Department, Instituto de Investigación Sanitaria Puerta de Hierro-Segovia de Arana, Madrid, Spain*
2. *Centro de Investigación Biomédica en Red de Cáncer (CIBERONC), Spain*
3. *Pathology Department/Translational Hematopathology Group, Hospital Universitario Marqués de Valdecilla/IDIVAL, Santander, Spain*
4. *Bioinformatics Unit, Spanish National Cancer Research Centre (CNIO), Madrid, Spain*
5. *CNAG-CRG, Centre for Genomic Regulation (CRG), Barcelona Institute of Science and Technology (BIST), Spain*
6. *Universitat Pompeu Fabra (UPF), Barcelona, Spain*
7. *Hematology Department, Hospital Universitario Puerta de Hierro-Majadahonda and Instituto de Investigación Sanitaria Puerta de Hierro-Segovia de Arana, Spain*
8. *Pathology Department, Hospital Fundación Jiménez Díaz, Madrid, Spain*

Figure S1. Patient 1: A) VAF of the non-synonymous somatic mutations. B) Clonal composition inferred by PyClone during tumor evolution. C) percentage of cells with cytogenetics alterations detected by FISH during disease progression. D) Clonal composition inferred by PyClone of different affected organs

A)

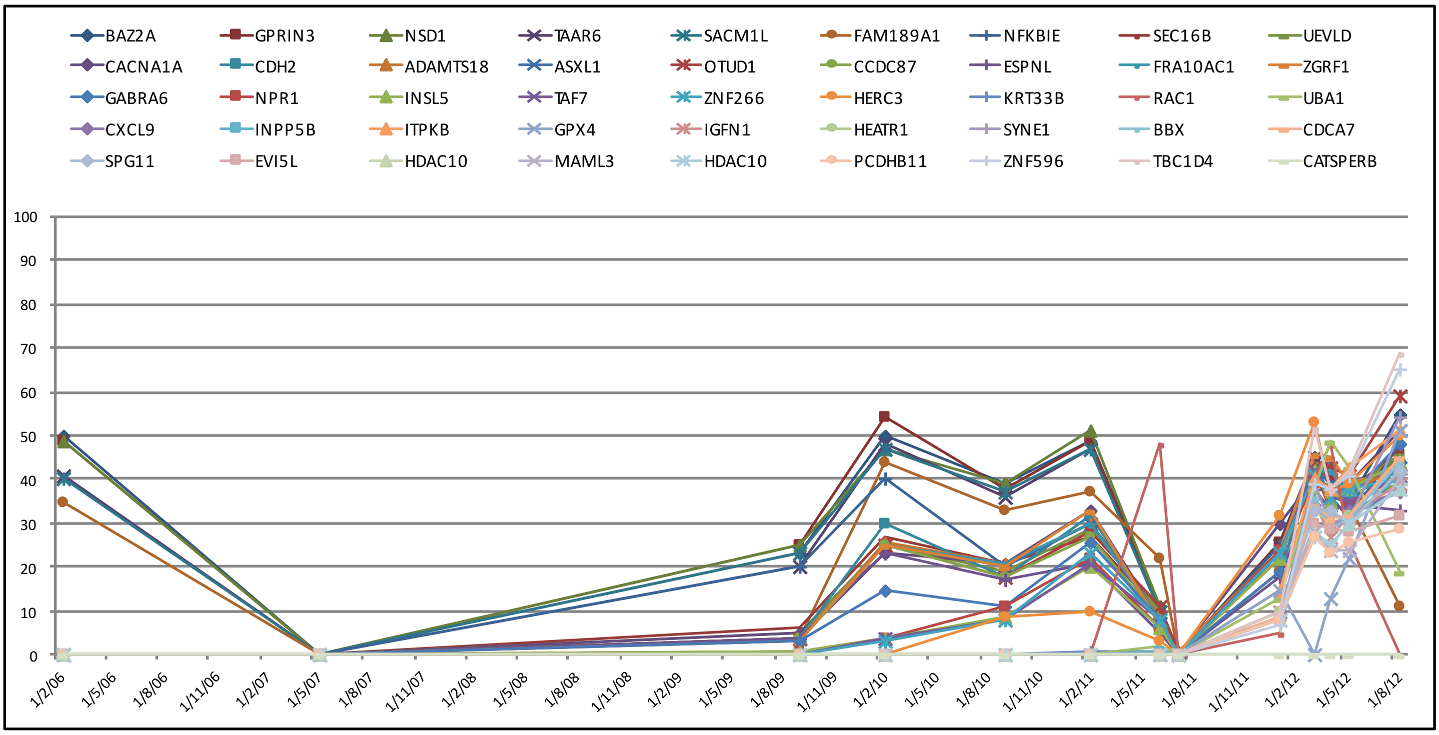

B)

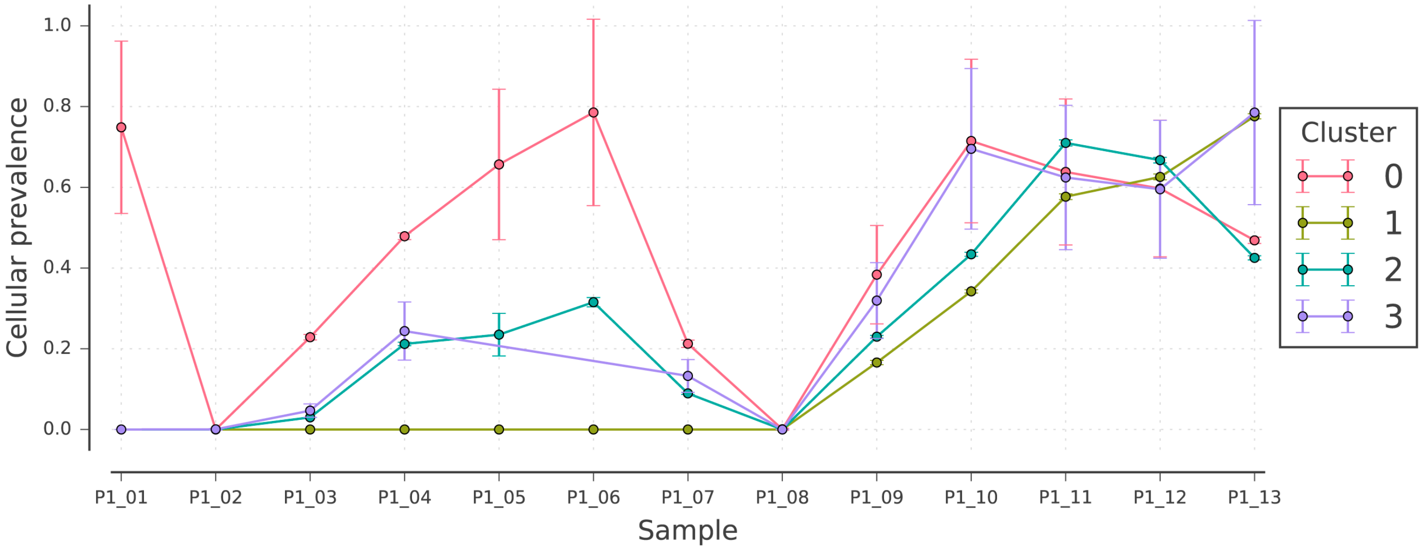

C)

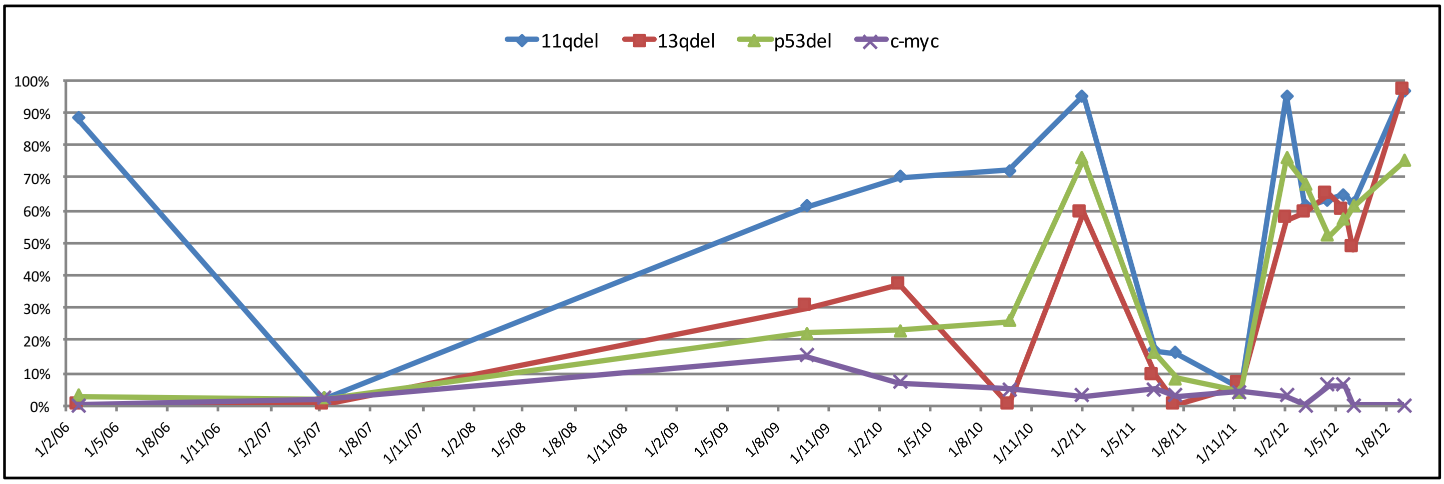

11qdel
13qdel
p53del
c-myc
100%
90%
80%
70%
60%
50%
40%
30%
20%
10%
0%
1/2/06
1/5/06
1/8/06
1/11/06
1/2/07
1/5/07
1/8/07
1/11/07
1/2/08
1/5/08
1/8/08
1/11/08
1/2/09
1/5/09
1/8/09
1/11/09
1/2/10
1/5/10
1/8/10
1/11/10
1/2/11
1/5/11
1/8/11
1/11/11
1/2/12
1/5/12
1/8/12

D)

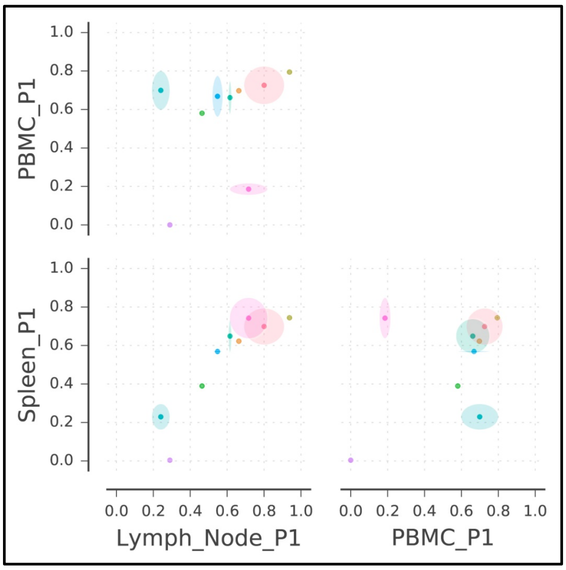

PBMC_P1
Spleen_P1
Lymph_Node_P1
PBMC_P1
0.0
0.2
0.4
0.6
0.8
1.0

Figure S2. Patient 2: A) VAF of the non-synonymous somatic mutations. B) Clonal composition inferred by PyClone during tumor evolution. C) percentage of cells with cytogenetics alterations detected by FISH during disease progression.

A)

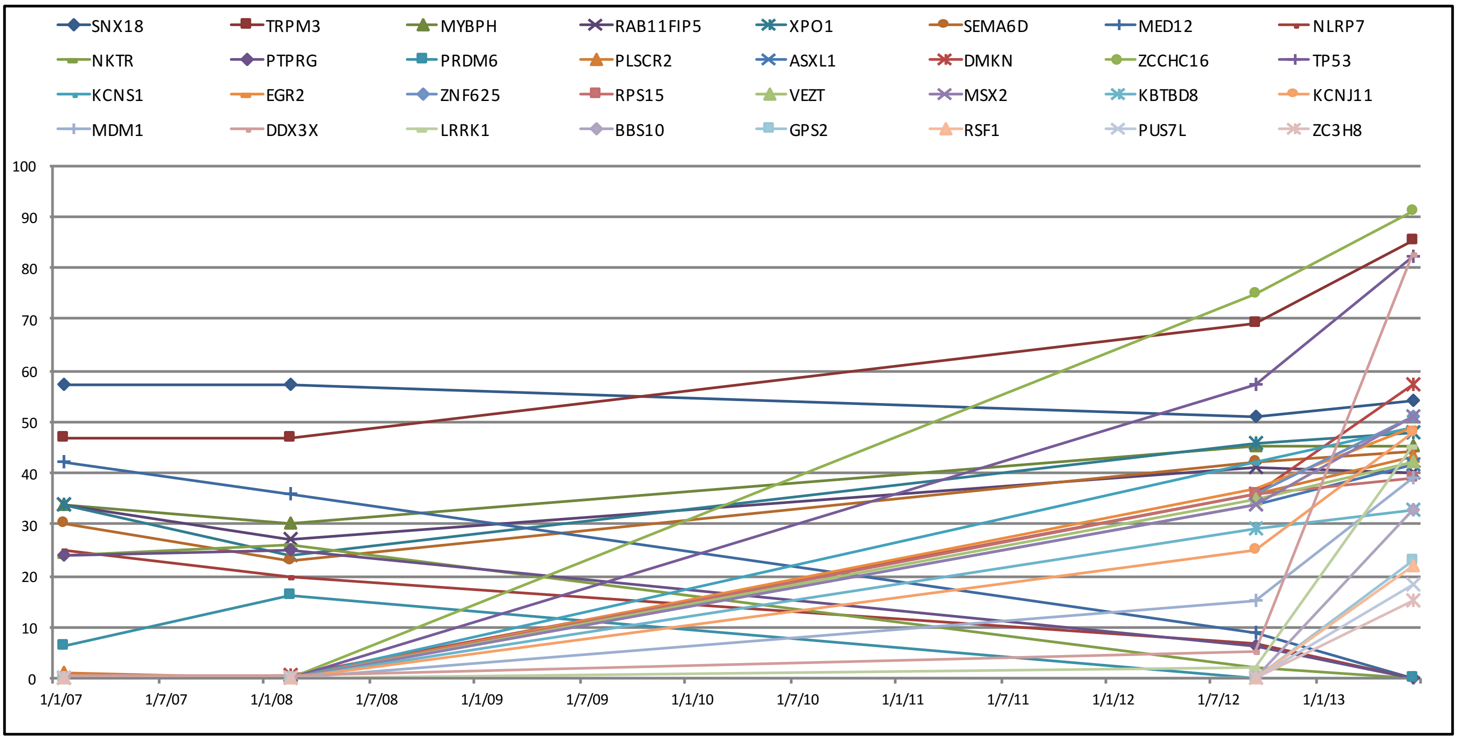

B)

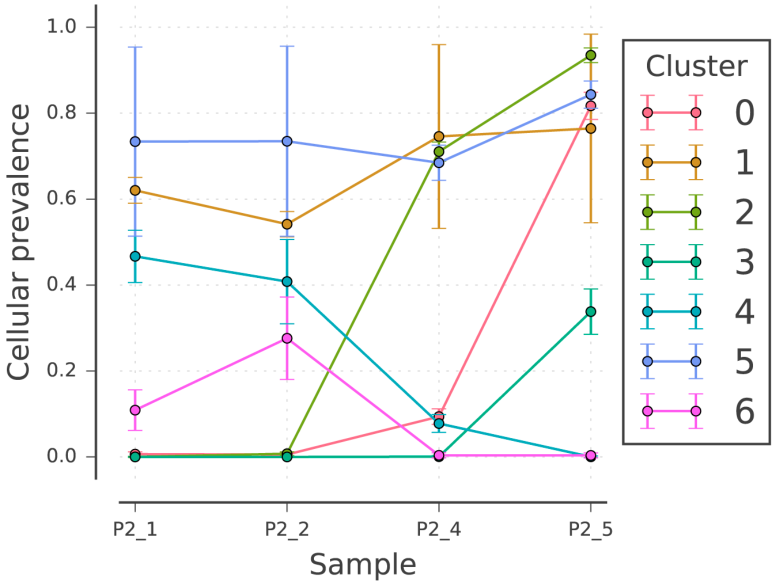

C)

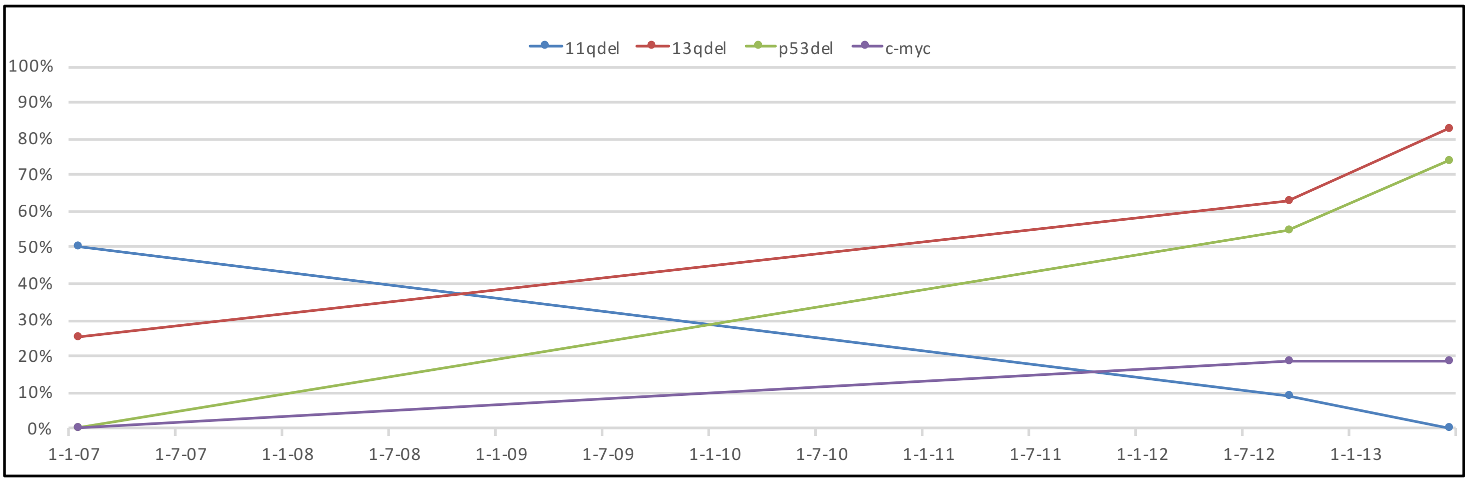
11qdel
13qdel
p53del
c-myc
100%
90%
80%
70%
60%
50%
40%
30%
20%
10%
0%
1-1-07
1-7-07
1-1-08
1-7-08
1-1-09
1-7-09
1-1-10
1-7-10
1-1-11
1-7-11
1-1-12
1-7-12
1-1-13
